# Hyperprolactinemia complicated with peliosis hepatis: One case report and review of literature

**DOI:** 10.1097/MD.0000000000041168

**Published:** 2025-01-10

**Authors:** Xun Li, Yang Lv, Yu Xia, Yulian Lai, Shangbin Chen, Yinzi Xie, Ziwei Tang, Qingfeng Cheng

**Affiliations:** a Department of Endocrinology, The First Affiliated Hospital of Chongqing Medical University, Chongqing, China; b Wuzhou Medical College, Wuzhou of Guangxi Zhuang Autonomous Region, Wuzhou, China.

**Keywords:** case report, hyperprolactinemia, liver dysfunction, peliosis hepatis, purpura liver disease

## Abstract

**Rationale:**

Peliosis hepatis (PH) is a rare disease with few clinical reports and complex etiology. However, there have been no reports of hyperprolactinemia (HPRL) leading to PH at present. This paper, through case reports, expands the understanding of the etiology of PH and the pathological damage effect of prolactin (PRL).

**Patient Concerns:**

The patient reported in this paper had jaundice, menstrual disorders, menopause, weight gain, and other symptoms. Laboratory examination found increased levels of PRL and transaminase in liver function, and imaging examination indicated pituitary tumor and PH.

**Diagnoses:**

Comprehensive patient history and auxiliary examination, the clinical diagnosis was pituitary PRL tumor and PH.

**Interventions and Outcomes:**

After treatment with bromocriptine, menstruation recovered and liver function returned to normal. In addition, the follow-up imaging examination indicated that pituitary tumors and PH lesions were shrinking, and clinical phenomena indicated that HPRL caused by pituitary prolactinoma was correlated with PH occurrence.

**Lessons:**

Since there is no report of HPRL causing PH, the specific pathogenesis is unknown. This paper reviews the relevant literature and puts forward the theoretical consideration of the pathogenesis. Through this case, for clinically similar patients, it is warned that we need to consider the possibility of PH and further improve the examination, evaluation and treatment in time.

## 1. Introduction

Hyperprolactinemia (HPRL) refers to a state in which the level of peripheral blood prolactin (PRL) is continuously increased due to various reasons, and its etiology can be classified into 4 categories: physiologic, pathological, pharmacologic, and idiopathic. High levels of PRL can cause a series of changes such as menstrual disorders or menopause, infertility, sterility, galactorrhea, and weight gain.^[1]^ Currently reported HPRL can be associated with hypothyroidism, osteoporosis, aldosteronism, and other diseases. However, there was no report of HPRL complicated with peliosis hepatis (PH).

## 2. Case data

### 2.1. General Information

Patient, female, 30 years old. She went to the hospital on July 11, 2023, due to “menstrual disorder for 2 years, menopause for 2 months, accompanied by weight gain and jaundice for 1 month.” Two years ago, the patient had no obvious cause of menstrual disorders, manifested as thin menstruation and small amounts. Besides, there has been no menstruation in the recent 2 months and a weight increase of about 10 kg in the recent 1 month, accompanied by edema of both lower limbs, sclera yellow stain, no fever, chills, headache, dizziness, visual field defect, and galactorrhea. The patient took Chinese medicine treatment 1 week ago, but it has had no therapeutic effect. Previous menstruation was regular, volume and color were normal, there were no blood clots, there was no dysmenorrhea, and the last menstruation time was May 1, 2023. Personal history: The patient was in good health. No abnormality was found in upper abdominal computed tomography (CT) during the physical examination 4 years ago, and liver function was normal during the physical examination 1 year ago. Marital history: Sexual life, unmarried, and without children. Family history: No patients with similar diseases in the family.

### 2.2. Physical examination

Height 162.00 cm, weight 58.00 kg, and body mass index 22.10 kg/m^2^. Clear mind, good spirit, physical examination cooperation, and answer the question. The sclera is mildly yellow, and the skin of the whole body has no yellow stain, no ecchymosis, no petechiae, no hairy acne, and no purple lines. Systemic superficial lymph nodes were not enlarged. The neck is soft, and the thyroid is not swollen. The thorax is symmetrical, the respiratory sounds of both lungs are clear, and the dry and wet rales are not heard. Heart boundary size was normal, heart rhythm was consistent, and no pathological murmurs were heard in the auscultation area of each valve. The abdomen was soft, the liver, spleen, and ribs were not touched, there was no tenderness, rebound pain, or muscle tension, and mobility dullness was negative. Physiological reflex was present, the pathological reflex was not elicited, and both lower limbs had pitted edema.

### 2.3. Auxiliary inspection

### 2.4. Treatment process

**Figure 1.**
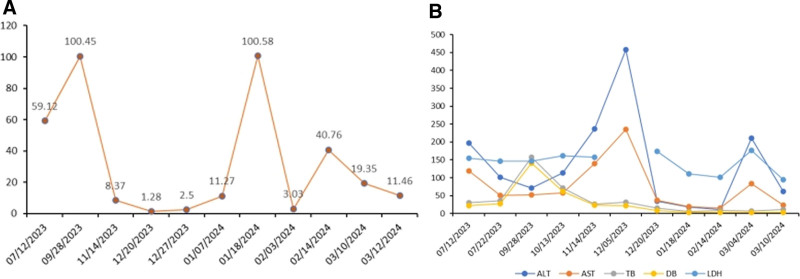
Index change curve. (A) Horizontal line chart of prolactin at different times. (B) Line chart of liver function changes at different times. ALT = alanine aminotransferase, AST = aspartate aminotransferase, DB = direct bilirubin, LDH = lumber disc herniation, TB = total bilirubin.

**Figure 2.**
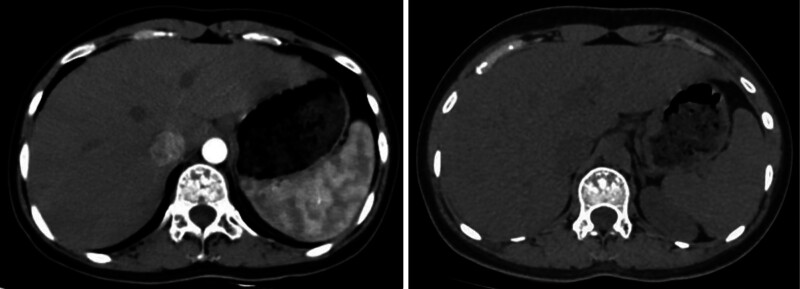
Abdominal enhanced computed tomography (CT) in July 11, 2020, and abdominal CT plain scan in July 21, 2023.

**Figure 3.**
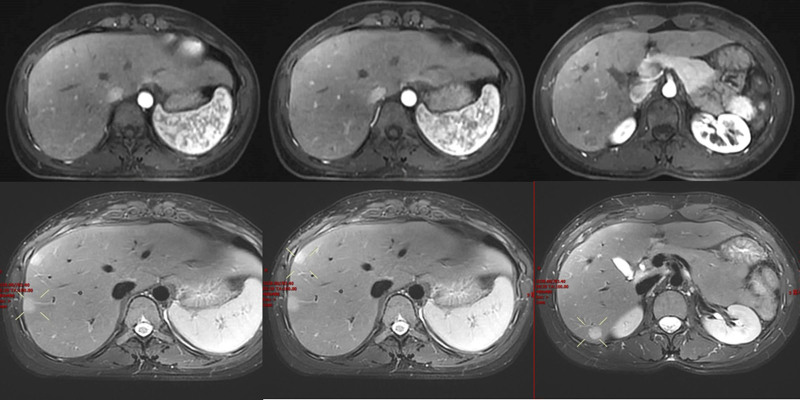
Upper abdominal magnetic resonance imaging in September 28, 2023: T1-weighted image arterial enhancement stage and T2-weighted image nonarterial enhancement stage.

**Figure 4.**
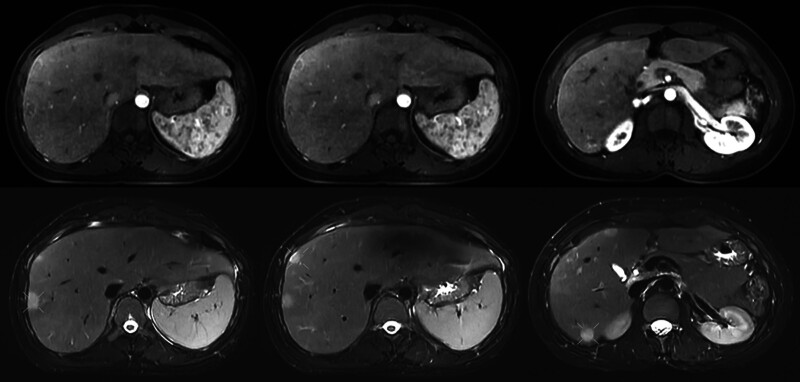
Upper abdominal magnetic resonance imaging with disodium gadosenate in November 18, 2023: T1-weighted image arterial enhancement phase and T2-weighted image nonarterial enhancement phase.

**Figure 5.**
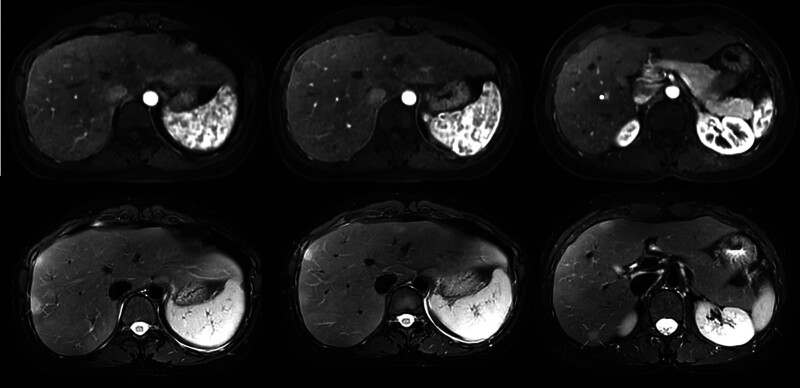
Liver 4Dflow magnetic resonance imaging in April 20, 2024: arterial enhancement phase on T1-weighted image and nonarterial enhancement phase on T2-weighted image.

**Figure 6.**
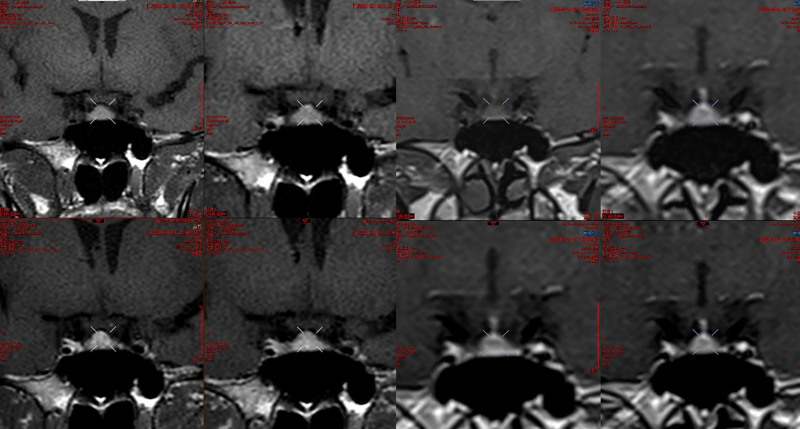
Comparison of pituitary magnetic resonance imaging enhancement between in October 1, 2023, and January 19, 2024.

On July 11, 2023, the patient had a history of taking Chinese medicine, so consider the disease as drug-induced liver injury or HPRL (Tables 1 and 2; Figs. 1–6). Ask the patient to stop using Chinese medicine. After 10 days (July 22, 2023), the liver function did not return to normal, and diammonium glycyrrhizinate capsules combined with polyene phosphatidylcholine were given for liver protection treatment. After 2 + months of follow-up (September 28, 2023), liver function still did not return to normal, the level of bilirubin was higher than before, and the level of PRL was higher than before after reexamination, to further improve the examination (Table 2), the diagnosis should be considered: HPRL, pituitary PRL tumor, and PH. After 2 weeks of temporary liver protection treatment (October 13, 2023), the liver function continued to be abnormal, and then bromocriptine mesylate was added at 5 mg/d, and menses appeared 1 month after treatment. After 2 months of treatment (December 20, 2023), PRL returned to normal, liver function returned to normal, and bromocriptine mesylate was adjusted to 1.25 mg/d for treatment. Three days after withdrawal (January 18, 2024), PRL increased again, and bromocriptine mesylate was given 5 mg/d. PRL decreased 1 month after treatment (February 14, 2024), and magnetic resonance imaging (MRI) enhancement of the pituitary gland during follow-up indicated that the lesion was slightly smaller than before. After 1 + month of treatment (March 10, 2024), PRL level was normal and adjusted to bromocriptine mesylate 1.25 mg/d maintenance therapy. During the follow-up, PRL levels continued to be normal, and abnormal transaminase appeared in liver function, which was considered to be related to taking drugs after respiratory tract infection at that time and returned to normal after stopping the drugs. In addition, after treatment, the liver MRI on April 20, 2024, follow-up showed that the PH lesions shrank, and the blood lipid returned to normal without taking lipid-lowering drugs (Table 1).

**Table 1:** Laboratory examination.

	July 12, 2023	July 22, 2023	September 28, 2023	October 13, 2023	November 14, 2023	December 5, 2023	December 20, 2023	January 18, 2024	February 3, 2024	February 14, 2024	March 4, 2024	March 10, 2024	March 12, 2024	May 20, 2024	Reference range
PRL (ng/mL)	59.12		100.45		8.37		1.28	100.58	3.03	40.76		19.35	11.46	24.49	Child-bearing period: 3.34–25.72, menopause: 2.74–19.64
ACTH (pg/mL)	16.03				35.40										7.00–65.00
GC (nmol/L)	550.15														124.20–662.40
FSH (ng/mL)	0.17		0.60		1.03		0.08						0.20		*
LH (mIU/mL)	<0.20		<0.20		0.40		0.08						0.05		†
E2 (pg/mL)	10.06		<15		11.60		13.58						11.64		‡
P (ng/mL)	0.74		0.45		1.46		0.35						0.58		§
T (ng/mL)	0.19		0.37		0.18		<0.10						<0.10		<0.75
TB (umol/L)	30.20	35.80	157.30	71.50	26.20	32.00	15.80	6.40		8.50	6.70	10.70		4.6	0–21.00
DB (umol/L)	22.30	27.10	140.80	61.40	23.50	21.80	8.50	3.70		3.40	3.10	3.40		1.5	0–8.00
ALT (U/L)	197.00	102.00	72.00	113.00	236.00	458.00	34.00	18.00		11.00	211.00	62.00		6	7.00–40.00
AST (U/L)	119.00	51.00	52.00	57.00	139.00	235.00	37.00	20.00		15.00	83.00	23.00		10	13.00–35.00
ALP (U/L)	39.00	43.00	52.00	53.00	40.00		51.00	34.00		36.00	39.00	34.00		32	35.00–100.00
GGT (U/L)	89.00	118.00	389.00	244.00	189.00	732.00	93.00	19.00		10.00	32.00	18.00		10	7.00–45.00
LDH (U/L)	154.00	146.00	146.00	161.00	157.00		174.00	111.00		102.00	177.00	94.00		92	120.00–250.00
TC (mmol/L)			12.13											4.06	2.80–5.20
TG (mmol/L)			4.08											1.39	0.35–1.70
HDL (mmol/L)			0.43											1.08	1.04–1.55
LDL (mmol/L)			6.36											2.63	0–3.37
Ga^2+^ (mmol/L)	2.11														2.15–2.50
Others	July 11, 2023	β-HCG: (-). Blood routine, urine routine, renal function, thyroid function, autoantibodies, and hepatitis B were not abnormal.													
	July 22, 2023	Coagulation function, fasting blood glucose, CA125, human epididymis protein4, antinuclear antibody profile, autoimmune liver disease detection, viral hepatitis 9 items, and ceruloplasmin were not abnormal.													

ACTH = adrenocorticotropic hormone, ALP = alkaline phosphatase, ALT = alanine aminotransferase, AST = aspartate aminotransferase, CA125 = carbohydrate antigen 125, DB = direct bilirubin, E2 = estradiol, FSH = follicle stimulating hormone, GC = glucocorticoid, GGT = gamma-glutamyltransferase, HDL = high density lipoprotein, LDH = lactate dehydrogenase, LDL = low-density lipoprotein, LH = luteinizing hormone, P = progesterone, PRL = prolactin, T = testosterone, TB = total bilirubin, TC = total cholesterol, TG = triglycerides, β-HCG = β-human chorionic gonadotrophin.

* Luteal phase: 1.79–5.12, ovulatory period: 4.54–22.51, follicular phase: 3.85–8.78, menopause: 16.74–113.59.

† Luteal phase: 1.2–12.86, ovulatory period: 19.18–103.03, follicular phase: 2.12–10.89, menopause: 10.87–58.64.

‡ Luteal phase: 30.34–274.24, ovulatory period: 29.42–442.62, follicular phase: 15.16–148.13, menopause: <38.90.

§ Luteal phase: 5.15–18.56, follicular phase: 0.31–1.52, menopause: <0.78.

**Table 2:** Imaging examination.

Time	Item	Content
July 21, 2020	Upper abdominal CT enhancement	Liver shape and size were normal; liver parenchymal scan and enhancement showed no abnormal shadow; gallbladder, pancreas, spleen shape, and size were normal; and parenchymal density was normal.
July 11, 2023	Epigastrial CT scan	Intrahepatic calcification; no abnormalities were found in the gallbladder, bile duct, and pancreas. Left adrenal punctate calcification, left renal small vessel leiomyoma.
September 28, 2023	Abdominal ultrasound	The liver echo is slightly dense and enhanced, less homogenous, with a liver calcification focus and high threshold of portal pulse; gallbladder, gallbladder polypoid lesion after drinking water; splenomegaly; and slightly hyperechoic nodules of the left kidney.
	Liver elastography	Liver hardness value: 6.9 Kpa.
	Gynecologic ultrasound	Uterine posterior position, size 105 mm×78 mm×81 mm, irregular shape, uneven myometral echo, intrauterine low echo, with a slightly higher echo between, size about 82 mm×33 mm, borderline unclear, CDFI showed blood flow signal inside, RI: 0.60.
	Upper abdomen + pelvic MRI enhancement	The size and shape of the liver were normal, the nodular T2 scattered in the liver parenchyma had slightly high signals, some nodules had blurred edges, ADC signals were not low, and the enhancement showed delayed intensification. The possibility of inflammatory lesions was considered. The liver bile duct, gallbladder, pancreas, and spleen were normal. Endometrial hyperplasia may be accompanied by polyp formation, a small amount of hematocele in the uterine cavity, and a small amount of fluid in the pelvic cavity.
October 1, 2023	Pituitary MRI enhancement	The pituitary is full in shape, the upper margin is swollen upward, and the dynamic reinforcement is not uniform in early, middle, and late stages, indicating the possibility of a pituitary tumor.
November 18, 2023	Upper abdominal MRI enhancement (disodium gadolinium)	Hepatosplenomegaly, scattered nodular abnormal signals in the hepatic parenchyma, slightly low signal on T1WI, slightly high signal on T2WI, lesions in the right lobe of the liver subcapsular, the length of the larger one is about 2.0 cm, enhanced fill-like enhancement, considering benign lesions, possible hepatic purpura, hepatic adenomatosis (inflammatory type)? No abnormalities were found in the bile duct, gallbladder, or pancreas. Bilateral adrenal atrophy, combined with anterior pituitary and uterine changes, not excluding hormonal endocrine changes.
January 19, 2024	Pituitary MRI enhancement	The pituitary gland was full in shape, slightly swollen upward at the upper margin, and the dynamic enhancement was uneven in early, middle, and late stages, and the lesion was slightly reduced compared with 2023-10-1.
January 23, 2024	Hysteroscope	Endometrial lesions, cervical polyps. Pathology (intrauterine tissue: endometrial polyps).
March 13, 2024	Bone mineral density	Osteopenia
April 20, 2024	Liver MRI enhancement (4Dflow)	The liver and spleen were enlarged, and nodular abnormal signals were scattered in the hepatic parenchyma, with low signals on T1WI and slightly high signals on T2WI. The lesions were mostly concentrated under the hepatic capsule, and the larger lesions were about 1.6 cm in length and continued to strengthen, which was slightly smaller than some lesions in the 2023-11-18MR film. No abnormalities were found in the bile duct, gallbladder, or pancreas. Small cyst of left kidney possible.

ADC = apparent diffusion coefficient, CDFI = color doppler flow imaging, CT = computed tomography, MRI = magnetic resonance imaging, RI = resistance index, T1WI = T1-weighted image, T2WI = T2-weighted image.

## 3. Discussion

**Figure 7.**
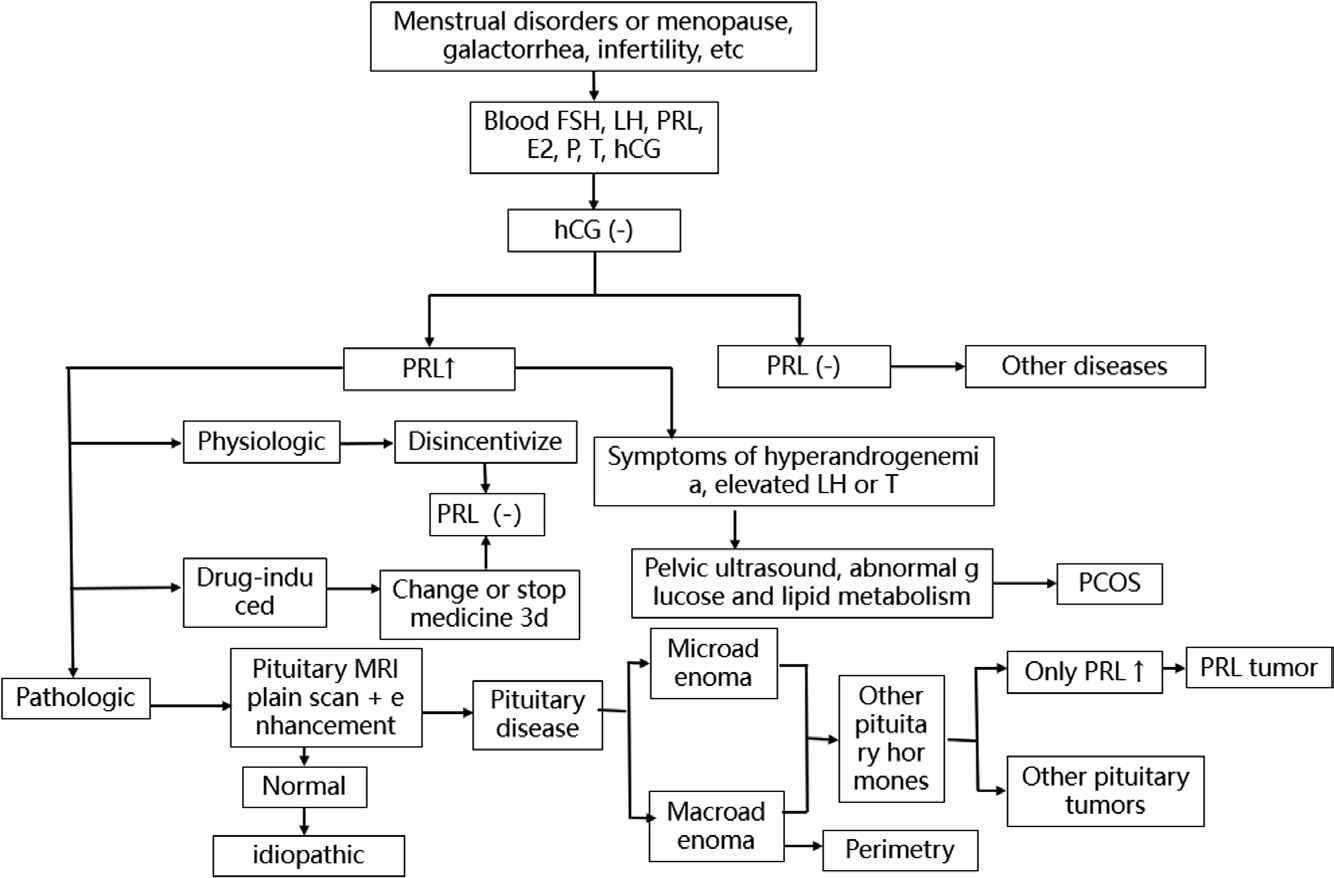
Hyperprolactinemia diagnosis process.^[4]^ E2 = estradiol, FSH = follicle stimulating hormone, hCG = human chorionic gonadotropin, LH = luteinizing hormone, MRI = magnetic resonance imaging, P = progesterone, PCOS = polycystic ovary syndrome, PRL = prolactin, T = testosterone.

HPRL is an increase in the level of PRL in peripheral blood caused by various reasons, which can cause dysfunction of the hypothalamic-pituitary-gonadal axis. The clinical manifestations are mainly oligomenorrhea or menopause, infertility, abnormal lactation, and some patients may be complicated with weight gain, bone pain or bone loss, hirsutism, seborrhea, acne, etc.^[2]^ It can also inhibit the excretion of kidney water and sodium, resulting in the formation of tissue edema.^[3]^ Its etiology includes 4 categories: physiologic, pharmacologic, pathological, and idiopathic,^[4]^ and the specific diagnosis process is shown in Figure 7. The patient was found to have abnormal PRL at the first visit. Due to the history of taking Chinese medicine, the influence of drug factors could not be ruled out at this time, so consider the disease and follow up. At the second visit, the level of PRL was significantly higher than before. Considering the possibility of pathological factors, the pituitary microadenoma detected by MRI enhancement was further improved. Combined with the patient’s menopause, weight gain, low gonadal axis hormone level, and bone loss, while the adrenocorticotropic hormone and cortisol levels were normal, the HPRL caused by pituitary PRL tumor was considered. After treatment with bromocriptine mesylate, the level of PRL decreased, and the MRI enhancement of the pituitary gland during follow-up showed that the lesion shrank and menses appeared, which further verified the clinical diagnosis of PRL tumor of the pituitary gland, and the PRL tumor of the pituitary gland was also an important factor causing HPRL.^[5]^ In addition, reviewing the PRL test of the patient, the level of PRL was slightly increased for the first time, and there may be a diagnostic defect of the PRL test—hook effect; that is, with the gradual increase of the concentration of analyte in the hormone test sample, the concentration curve rises, and when the hormone concentration is higher than a certain critical point, the measurement curve drops like a hook, and the measurement result is significantly lower than the actual level.^[6]^

**Figure 8.**
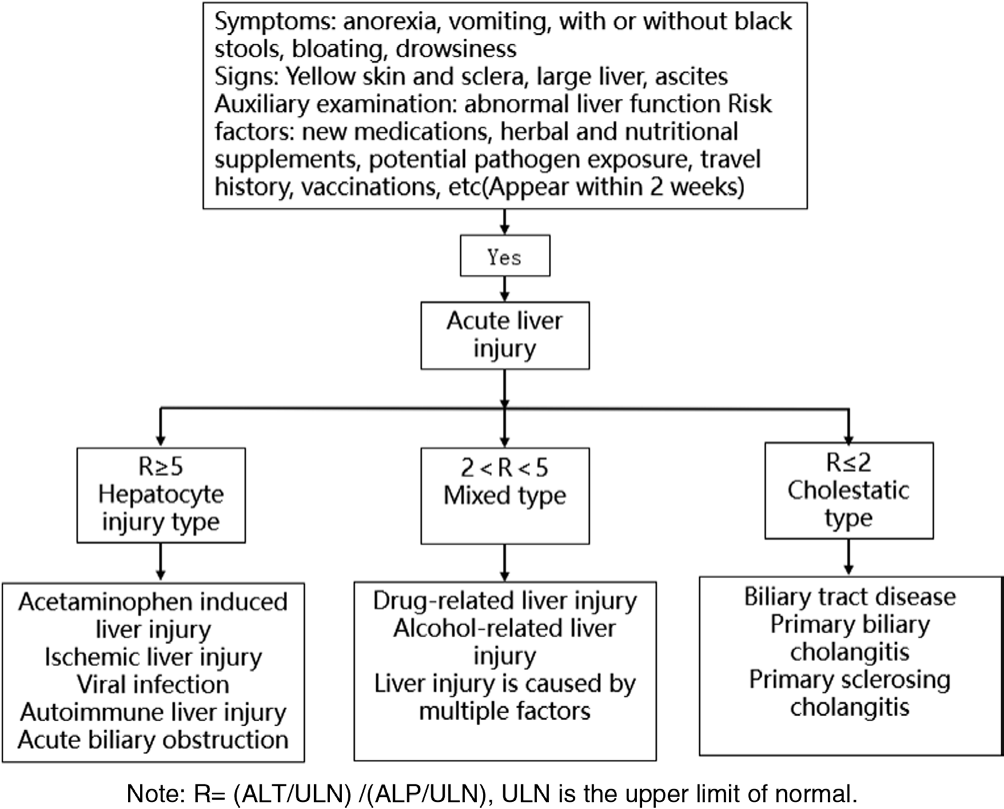
Diagnostic process of acute liver injury.^[7]^ ALT = alanine aminotransferase, ALP = alkaline phosphatase.

The special feature of this patient is that in addition to HPRL, liver function injury is also complicated, and it can be divided into hepatocellular injury, mixed type, and cholestasis type according to *R* value^[7]^ (Fig. 8.). The *R* value at the first visit was 12.63, and because of the history of taking Chinese medicine, the drug-induced hepatocellular injury was considered. However, after the withdrawal of Chinese medicine, the liver function continued to be abnormal with *R* value still >5, and the liver function did not return to normal after the treatment with hepatoprotective drugs, but there was a phenomenon of bile enzyme separation. The common cause of bile enzyme separation was massive necrosis of hepatocytes and decreased ability of surviving hepatocytes to process bilirubin. In addition, obstructive jaundice can also lead to the separation of cholase,^[8]^ but the patient’s blood routine and biliary system examination showed no abnormalities, so it was considered that the cause was related to hepatic parenchymal lesions, and combined with enhanced MRI of the upper abdomen, benign intrahepatic lesions—PH—were suggested. PH, also known as purpura liver disease, is a rare type of solid space-occupying lesion of the liver, which was first discovered by Wagner in 1861 and named by Schoen lank in 1961. Its pathological manifestations include multiple congested small cystic cavities in the liver.^[9]^ According to the liver involvement, it can be divided into focal type and diffuse type. The clinical manifestations and laboratory tests of the PH lack specificity: cholestasis (skin or sclera yellow staining), hepatosplenomegaly, hepatic percussion pain, mobile dullness, and edema of both lower extremities may occur clinically. In severe cases, lesions rupture bleeding, hemorrhagic shock, liver failure, and hepatic encephalopathy may occur. In laboratory tests, elevated aminotransferase, bilirubin, and lumber disc herniation may be found in some patients, while no abnormal changes in liver function can be found in some patients.^[10,11]^ Although imaging examination can provide some clues for the diagnosis of the PH, it also lacks specificity. The lesions are mostly hypoechoic on abdominal ultrasound, and some of them are complicated with enlarged liver or spleen and thickened liver echo. On enhanced CT, there were mostly low-density lesions, which showed mild uneven enhancement after enhancement, mostly with low central density. In the portal phase, the lesions showed “centripetal” or “centrifugal” enhancement. In the delayed phase, the density of the lesions was gradually uniform, and the density was higher or lower than that of the surrounding tissues. On MRI, T1 low signal, T2 high signal, and annular enhancement in the arterial phase, the typical “target sign” can be seen, and the lesions in the delayed phase show “centered” enhancement,^[12,13]^ but it needs to be differentiated from liver cancer, hepatitis pseudotumor, hepatic hemangioma, focal hyperplastic nodules of the liver, and other diseases.^[11]^ Combined with this case, the clinical manifestations of the patient were jaundice and edema of both lower limbs; laboratory examination indicated elevated levels of transaminase and bilirubin; MRI enhancement of the upper abdomen with disodium gadolinite was consistent with PH findings, and the liver and spleen were enlarged, so the clinical diagnosis was PH. However, the etiology of PH is not clear at present, and some scholars have proposed some possible etiology^[10,14]^ (Table 3), involving various factors such as immunity, tumor, infection, and drugs. The pathogenesis also includes many theories: 1 is congenital vascular malformation. Second, the dilatation of hepatic sinuses was caused by obstruction of the junction of hepatic sinuses and central veins. The third is the formation of a cystic cavity after focal necrosis of hepatocytes. Fourth, the damage to the hepatic sinus wall was caused by toxic substances. Fifth, the destruction of reticular scaffolds led to the expansion of hepatic sinus space.^[9,15]^

**Table 3:** Causes of hepatic purpura.^[10,14]^

1. Blood system
Multiple myeloma, Hodgkin disease, leukemia, lymphoma, lymphosarcoma, aplastic anemia, paroxysmal sleep hemoglobinuria, globular erythrocyte hemolysis, Fanconi anemia, and macroglobulinemia
2. Urinary system
Kidney transplantation, renal cell carcinoma, pyelitis, pyelitis, glomerulonephritis, and long-term hemodialysis
3. Digestive system
Liver benign tumor, hepatocellular carcinoma, liver abscess, acute hepatitis, chronic alcoholic liver disease, intrahepatic cholestasis, oral inflammatory diarrhea, gastric cancer, colorectal cancer, and chronic pancreatitis
4. Cardiovascular system
Heart failure, acute myocardial infarction, heart transplantation, and polyarteritis
5. Respiratory system
Lung cancer and spontaneous pneumothorax
6. Nervous system
Parkinsonism
7. Endocrine system
Diabetes, breast cancer, and thymus tumors
8. Bone and joint diseases
Rheumatoid arthritis and osteoporosis
9. Infectious diseases
Tuberculosis, leprosy, suppurative shock, herpes, and bacterial endocarditis
10. Others
Malnutrition and acquired immunodeficiency disease
11. Medication
Androgenic hormone, contraceptive, adrenal corticosteroid, azathioprine, methotrexate, tamoxifen, arsenic, thorium dioxide, vinyl chloride, and copper sulfate
12. Idiopathic purpura of unknown cause

Further analysis of the etiology and pathogenesis showed that the patient had no history of PH-related diseases, and no abnormalities were found in liver-enhanced CT and liver function examination, which excluded congenital vascular malformation from the pathogenesis. The patients stopped using Chinese medicine immediately after liver function impairment was found, and the liver function continued to be abnormal after subsequent treatment with hepatoprotective drugs. Combined with the clinical manifestations of cholestasis before taking Chinese medicine, drug toxicity was ruled out. In addition, under HPRL standard treatment, liver function gradually returned to normal and hepatic purpura lesions shrank, indicating that HPRL was correlated with the PH. However, HPRL has not been reported to cause the PH, so the specific pathogenesis is unknown. Only the following can be inferred: first, combining the *R* value of the liver function of patients to indicate hepatocyte injury, the pathogenesis is speculated to be related to hepatocyte necrosis. Previous studies have reported that PRL secreted into the circulatory system can regulate energy metabolism balance in a variety of tissues, such as the liver, brain, pancreas, and adipose tissue. In the liver, PRL level under physiological conditions has a protective effect. However, in the state of pathological elevation, the activity of the CYP3A4 enzyme can be increased, resulting in liver insulin resistance, triglyceride deposition, and increased adipogenesis, which is related to dyslipidemia, nonalcoholic fatty liver disease, and other diseases.^[16]^ In addition, the patient does have dyslipidemia, and abdominal ultrasound indicates that the slightly dense and enhanced liver echo is a manifestation of hepatic steatosis.^[17]^ At the same time, this metabolic abnormality is alleviated after the etiological treatment, which coincides with the treatment principle of PH. Perhaps PRL causes mitochondrial damage and lipid deposition in hepatocytes through the regulation of liver enzyme activity. Further progression to steatosis and necrosis in cases where the cause is not corrected leads to the development of PH. In addition, the PRL molecule contains 3 disulfide bonds, and its receptor PRLR is highly expressed in the liver.^[18]^ Meanwhile, the hepatocyte toxicity of azathioprine binding is correlated with PH (Table 3), so it may be that pathologically elevated PRL causes cell damage and necrosis after accumulation in the liver. Second, considering that PRL molecules in serum mainly exist in 3 different forms, namely monomer PRL (23 kD), large PRL (40–60 kD), and macroprolactin (M-PRL) molecule (>150 kD) bound to immunoglobulin G(IgG),^[19]^ When the serum M-PRL concentration exceeds 60% of the total PRL concentration, macroprolactinemia is present,^[20]^ and the prevalence of M-PRL in HPRL patients can be as high as 35%.^[21]^ This patient cannot rule out the possibility of complicating M-PRL. Due to the large molecular weight of M-PRL, there is the possibility of accumulation and deposition in blood vessels, resulting in embolization of the outflow tract of hepatic sinuses, that is, obstruction of the junction of hepatic sinuses and central vein, leading to expansion of hepatic sinuses, and then the occurrence of PH.

The clinical manifestations and auxiliary examination of PH lack specificity, and the gold standard for diagnosis is liver pathological biopsy. However, due to the high risk of bleeding during and after puncture, it is necessary to be cautious in clinical practice, so the diagnosis of PH is difficult. The patient did not undergo a liver biopsy and could only be clinically diagnosed with “PH” based on historical data. The treatment principles of PH include elimination of inducement, symptomatic treatment, regular follow-up, prevention of complications, etc. When the lesion is < 5 cm and asymptomatic, no intervention can be temporarily performed for follow-up observation. When the lesion is > 5 cm, microwave and radiofrequency ablation techniques can be used to treat the lesion. When the lesion is large and has obvious compression symptoms or the single lesion has a tendency of rupture and bleeding, it is feasible to resect the lesion. When the lesion is diffuse and accompanied by liver failure, orthotopic liver transplantation or liver transplantation is feasible.^[22]^ This patient had focal PH, and the largest lesion was 2.0 cm. Due to the combination of jaundice, liver function impairment, and liver and spleen enlargement, the primary disease, HPRL, was actively treated and was relieved after treatment, which once again verified the clinical diagnosis of PH.

PH is a rare disease with few clinical reports, and there has been no report of HPRL causing PH. Through this case report, our understanding of the cause of PH has been expanded. However, the limitation of this report lies in the lack of molecular detection of M-PRL and pathological biopsy, resulting in insufficient clinical diagnostic evidence and only theoretical analysis of the pathogenesis. For patients with clinically similar diseases, the PEGylated precipitation method can be used to detect M-PRL molecules,^[19]^ and liver pathological biopsy can be improved when necessary to confirm the diagnosis and pathogenesis.

Table 3. Causes of peliosis hepatis^[10,14]^

## Author contributions

**Writing—original draft:** Xun Li, Yang Lv.

**Investigation:** Yu Xia, Yulian Lai, Shangbin Chen.

**Visualization:** Yu Xia, Yulian Lai, Shangbin Chen.

**Data curation:** Yinzi Xie.

**Writing—review & editing:** Ziwei Tang, Qingfeng Cheng.
